# The year of transparency: measuring quality of cardiac care

**DOI:** 10.1007/s12471-015-0739-9

**Published:** 2015-08-14

**Authors:** D.C. Eindhoven, E. Wierda, M.C. de Bruijne, G. Amoroso, B.A.J.M. de Mol, V.A.W.M. Umans, M.J. Schalij, C.J.W. Borleffs

**Affiliations:** 1 Department of Cardiology, Leiden University Medical Center, PO Box 9600, 2300 RC Leiden, The Netherlands; 2 Department of Cardiology, Onze Lieve Vrouwe Gasthuis, Amsterdam, The Netherlands; 3 Department of Public and Occupational Health, EMGO Institute for Health and Care Research, VU University Medical Center, Amsterdam, The Netherlands; 4 Department of Cardiothoracic Surgery, Academic Medical Center, Amsterdam, The Netherlands; 5 Department of Cardiology, Medical Center Alkmaar, Alkmaar, The Netherlands

**Keywords:** Quality of healthcare, Quality indicators, Cardiovascular outcomes, Transparency

## Abstract

The assessment of quality of care is becoming increasingly important in healthcare, both globally and in the Netherlands. The Dutch Minister of Health declared 2015 to be the year of transparency, thereby aiming to improve quality of care by transparent reporting of outcome data. With the increasing importance of transparency, knowledge on quality measurement will be essential for a cardiologist in daily clinical care. To that end, this paper provides a comprehensive overview of the Dutch healthcare structure, quality indicators and the current and future assessment of quality of cardiac care in the Netherlands.

## Introduction

The assessment of quality of care is becoming increasingly important in healthcare, both globally and in the Netherlands. With the transition into a regulated healthcare market system in 2006, insurance companies received a central role and the shared legal responsibility for the quality of cost-effective care. This responsibility created the legal need to develop a system in which quality of care can be measured and monitored [1]. Currently, hospital accreditation is already based on quality measurements and in the future, reimbursement will most likely be based on quality instead of price and volume only [2]. The Dutch Minister of Health has declared the year 2015 to be the year of transparency, thereby stressing the need for reporting of measurable quality of care [3, 4]. With the increasing importance of transparency, knowledge on quality measurement will become vital in daily clinical care. The current manuscript provides a comprehensive overview of the Dutch healthcare structure, quality indicators and the current and future assessment of quality of care in the Netherlands.

### Definition of quality indicators

**Fig. 1:**
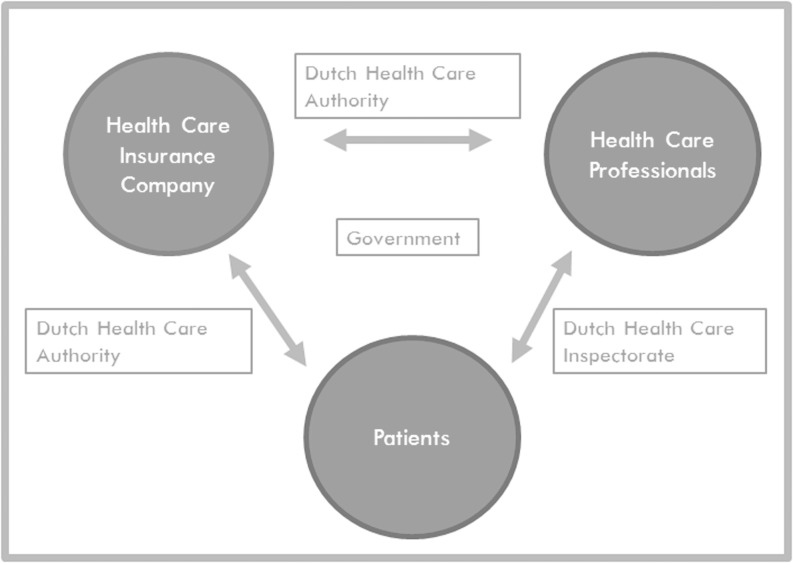
Different positions in the health care system in the Netherlands

Quality of care has been defined by the Agency for Healthcare Research and Quality of the United States as *doing the right thing, at the right time, in the right way, for the right person—and having the best possible results* [5]. The aim of measuring quality of care changes with the different positions in the healthcare system [6]. Patients aim for the best possible outcome and need quality measurements to be able to take informed decisions. Healthcare professionals aim for the best possible outcome for a maximum number of patients and, additionally, need quality measurements to benchmark results with other healthcare professionals in order to identify room for improvement. Healthcare insurance companies aim for the best possible (long-term) value for the money spent on behalf of their customers (insured patients). The government aims to achieve the best possible public health at a stated budget, while guaranteeing financial and physical accessibility and affordability for all inhabitants ([7]; Fig. 1).

To accomplish all these goals, quality of care has to be measured. In 1966, Donabedian described three different types of measurable indicators for quality of care: structure, process and outcome indicators ([8]; Tab. 1).

**Table 1:** Advantages and disadvantages of outcome, structure and process indicators

	Structure indicators	Process indicators	Outcome indicators
**Example**	*PCI volume a year*	*Medical prescription according to guidelines*	*Morbidity and mortality*
	*Availability of cathlab*	*Door-to-balloon-times*	*Functional health status*
	*Education level of the nurses*		*Patient satisfaction*
			*Costs*
**Advantages**	Appropriate	Reflect care that patients actually receive	The ‘bottom-line’ of cardiology
	If associated with outcome, inexpensive proxies of cardiological outcomes	Actionable from provider perspective	Outcomes measurement alone may improve outcomes
		Clear link to quality improvement activities	
**Disadvantages**	Most variables not actionable from provider perspective	Little information about which processes are important for specific procedures	Numbers too small to measure with adequate procedure-specific outcomes for most hospitals and procedures
	Imperfect proxies for outcomes reflect average results for large groups of providers, not individuals		Outcome measures that are not procedure-specific less useful for purposes of quality improvement

Based on table from Birkmeyer (2004) which is applied on examples from Cardiology [43].

*Structure indicators* reflect the system and setting in which care is delivered and measurements relate directly or indirectly to staff expertise or the organisation. For cardiac care, examples are PCI volume, availability of a catheterisation laboratory and the educational level of the nursing staff. Structure indicators are less likely to be influenced by medical professionals and therefore less useful to monitor programs for quality improvement. They reflect the average results for large groups of providers, not individuals. The advantage of these structure indicators is that they are expedient and inexpensive to collect and can be used in plain hospital comparisons. Structure indicators are in general of limited use in clinical practice although recently a large study (*n* = 457,498) was published in which a relationship was found between increased operator/institutional volume of PCI procedures and a decrease in adverse outcomes and cost of hospitalisation [9]. However, other studies demonstrated that an increase of volume above a certain threshold is not related to improved outcomes, hence some of these structure indicators may be useful to define minimal requirements.

*Process indicators* describe the care patients actually receive. Examples for cardiac care are door-to-balloon-times in patients with a ST-segment elevation myocardial infarction and medication prescription according to the guidelines [10, 11]. The usefulness of process indicators and the association with clinical outcome measures has been thoroughly established. In patients with myocardial infarction, Peterson et al. showed a correlation between processes of care and outcome. With every 10 % increase in process adherence (for example medication use according to guidelines) there was an associated 10 % decrease in in-hospital mortality [10]. Another study demonstrated 6 % of hospital-level variation of 30-day mortality rate to be explained by the performance on process measures [12]. In heart failure, the relationship between process and outcome is however modest. In the OPTIMIZE-HF study, none of the process measurements were associated with a decrease in 60- or 90-day mortality [13]. In case of a proven association, process indicators can be useful to monitor if aspects of clinical practice result in an improvement of the quality of care. A limitation, however, is that evidence on which processes are important for specific procedures is scarce. Importantly, although the use of process indicators is known to be effective in general, they do not mark the quality of care in individual patients. For example, patients with symptomatic bradycardia after myocardial infarction should not receive a beta-blocker, stressing the need for a connection with clinical data, which is more time-consuming.

Quality of care is most effectively measured by clinical outcome measures, referring to the effect of the provided care on the health status of patients: *outcome indicators.* Examples of these are overall mortality rate, hospital readmission rate, functional health status and patient satisfaction. Outcome measurement is considered the most important measurement of quality of care but has to be acquired per patient and is therefore relatively time-consuming and expensive. In 2013, the Court of Audit (Algemene Rekenkamer) concluded that the quality of most indicator sets is limited and that only 7 % of the indicators collected by hospitals were outcome indicators [14].

### Registration in cardiology in the Netherlands

#### National quality measurement

National quality measurements are initiatives from government, supervision institutions, insurance companies and patient organisations. From the perspective of the individual hospital and/or cardiology department these initiatives can be judged to be external requests for accountability.

The *Dutch Healthcare Inspectorate* (Inspectie voor de Gezondheidszorg, IGZ) has an important task, as described in Article 36 of the Healthcare Insurance Act, to verify if hospitals meet the minimum level of quality according to general healthcare acts and the professional standards as defined by the different medical specialists [4]. Verification is achieved by surveillance of compliance to the law, regulations, professional standards and guidelines. The Dutch Healthcare Inspectorate focuses on surveillance of the highest risks by mostly collecting process and structure indicators as delivered by the healthcare providers. For ST-segment elevation myocardial infarction, outcome, structure and process indicators (number of PCI procedures, in-hospital or 30-day mortality, door-to-needle time or door-to-balloon time and the percentage of patients referred for cardiac rehabilitation) are acquired. For pacemaker and implantable cardioverter defibrillator implantations the number of procedure-related complications within 90 days has to be registered [15]. The Safety Management System (Veiligheidsmanagementsysteem, VMS) is a Dutch patient safety program started by the Ministry of Health, Welfare and Sport and supported by all hospitals, primarily initiated to reduce avoidable patient injuries during hospital admission. These VMS indicators are now (partly) incorporated in the indicators collected by IGZ.

Hospitals use external *accreditation programs* to prove and objectify a certain level of quality of care as well as maintenance of quality of care to outsiders. The Netherlands Institute for Accreditation in Healthcare (Nederlands Instituut voor Accreditatie in de Zorg, NIAZ) aims to assure and improve Dutch healthcare by using an international accreditation program in which amongst others the VMS indicators are embedded. Some hospitals in the Netherlands use the international accreditation program, such as the Joint Commission International. Besides quantitative quality indicators, the accreditation systems comprise explicit quality policies and quality instruments, such as incident reporting and audits.

As described in the introduction, the Dutch healthcare system changed in 2006, aiming to reduce rising healthcare costs while improving quality of care. In order to achieve this, the Dutch government introduced a regulated healthcare market. Two new important acts were introduced: the Healthcare Insurance Act (Zorgverzekeringswet) and the Act of Regulation of Healthcare (Wet Marktordening Gezondheidszorg) [4, 16]. In the new system the *health insurance companies* play a central role, positioned between patients and caregivers, with a shared responsibility to ensure good quality and cost-effective care. For the first time it became possible for the insurance companies to selectively contract. Additional to the responsibility in limiting the rising healthcare costs, insurance companies are required to analyse and interpret quality of care provided by caregivers. Article 14 of the Healthcare Insurance Act, and the general directorial based on this, states that insurance companies share the responsibility for efficient and timely healthcare of good quality, based on professional standards defined by the scientific professional organisations and healthcare providers. The explanatory memorandum of the act states that more information on outcome of caregivers will be available in the future [17]. Currently, however, more attention is given to the volume and cost agreement than to the provided quality of care [18, 19]. The *Dutch Healthcare Authority* (Nederlandse Zorgautoriteit, NZa), with its task of overseeing the regulated healthcare market, is positive about the increased attention to quality of care in contracting during recent years [20]. A recent report of the Council for Public Health and Healthcare (Raad voor de Volksgezondheid en Zorg, RVZ) concluded that health insurance companies have to be more transparent about the criteria used for contracting care, which caregivers are contracted and how patients were involved in the process of contracting [21].

#### National quality registries in cardiology

When focussing on cardiology in the Netherlands, there are three large national registries: (1) the National Cardiovascular Data Registry (NCDR), (2) the Supervisory Committee for Heart Interventions in the Netherlands (Begeleidingscommissie Hartinterventie Nederland, BHN) and (3) Meetbaar Beter. These registries are all initiated by the healthcare professionals involved and funded by the participating hospitals. From the perspective of the hospital and cardiology department, these initiatives can be regarded as internal quality initiatives, primarily meant to improve the internal quality of the individual healthcare provider. Increasingly, on request of the government, Healthcare Inspectorate, insurance companies and patient organisations, these registers are used for external accountability as well. The NCDR was initiated by the Netherlands Society of Cardiology (Nederlandse Vereniging voor Cardiologie, NVVC) and organised in steering committees to develop different databases for every area in cardiology [22]. Furthermore, the NCDR data are sent to the national Implant Register (Medisch Implantatenregister), recently initiated by the Ministry of Health, Welfare and Sport [23]. The NCDR is NEN7510 certified, an information security certificate. Currently, data of more than 250,000 device patients are registered and almost all hospitals (85) participate in NCDR. The BHN, a collaboration of cardiothoracic surgery, cardiology, anaesthesiology and paediatric cardiology, is a national registry of cardiac interventions. The BHN includes data of all 16 cardiothoracic centres since 2007 [24]. Meetbaar Beter, initiated by the Catharina Hospital, Eindhoven and the St. Antonius Hospital, Nieuwegein, is a collaboration of currently 12 cardiothoracic centres in the Netherlands. In the near future Meetbaar Beter will also include data from PCI centres without on-site heart surgery. The Meetbaar Beter initiative raises a new Dutch concept of transparency reporting: a patient-oriented and physician-driven registry. Meetbaar Beter registers patient-outcome data in order to optimise clinical processes. Annually, these outcome indicators are analysed and when required, improvements are established [25]. Besides these national initiatives, in 16 out of 25 regions in the Netherlands, cardiologists collaborate with general practitioners, emergency services and patient representatives in a regional context which is called NVVC Connect. NVVC Connect aims to optimise regional care for myocardial infarction, atrial fibrillation and heart failure patients [26].

#### International registries

The first initiatives to register the quality of cardiac care on a national basis were started in Sweden and the United States. Currently, the Swedish registry SWEDEHEART collects data from all 74 hospitals in Sweden. In the United States, the American College of Cardiology (ACC) initiated the National Cardiovascular Data Registry (NCDR®CathPCI), which contains information on 12 million patients from 1577 participating centres [27]. England and Wales collect information in the Myocardial Ischaemia National Audit Project (MINAP)/National Institute for Cardiovascular Outcomes Research (NICOR) database on all patients with acute coronary syndrome, which contains data from all 236 hospitals [28, 29]. These registries are useful instruments for addressing important clinical questions by retrospectively selecting patients for a randomised trial [30]. An overview of other international AMI registries is given in Tab. 2. It emphasises that sufficient funding is important to ensure a solid registry.

**Table:** 

Country	National registry	Founded	Remarks
Belgium	Belgian STEMI project (44, 45) *BIWAC, Belgian Interdisciplinary Working Group on Acute Cardiology*	2007	Covering: obligatory for all Belgian hospitals; 50-60% STEMI patients a year are registrated Details: 3000 patients a year Variables: 25-30 Funding: Public, not linked with reimbursement
	PCI registry (46) *BWGIC, Belgian Working Group Interventional Cardiology*		Covering: all PCI hospitals Funding: Public, linked with a minimal reimbursement on PCI material
England/Wales	MINAP (28, 29) *Myocardial Ischaemia National Audit Project*	2000	Covering: all 236 acute hospitals in England and Wales for ACS patients (STEMI and NSTEMI) Details: 735 000 patients (2010) Variables: 123 Funding: Public, by participating hospitals
France	FAST-MI (47, 48) *French registry of Acute ST-segment elevation or non-ST-segment elevation Myocardial Infarction*	2005	Covering: 223 centres (60%). Data collection every five years Details: 1714 STEMI patients Variables: 385 in 2010 Funding: Public and private, by French Society of Cardiology and several pharmaceutical companies
Sweden	SWEDEHEART (28, 49) *A collaboration (since 2008) of RISK-HIA, SEPHIA, SCAAR, Swedish Heart Surgery Registry and Percutaneous Valve Registry*	2008	Covering: all 74 hospitals in Sweden for ACS patients undergoing CAG/PCI, percutaneous valve replacement or heart surgery. Details: 80.000 new patients each year (3 million in total) Variables: 106 variables ACS, 75 variables regarding secondary prevention, 150 variables for patients undergoing coronary angiography/angioplasty, 100 variables heart surgery. Funding: Public, by the Swedish Association of Local Authorities and Regions. Not linked with reimbursement
Switzerland	AMIS Plus (50) *Acute Myocardial Infarction in Switzerland*	1997	Covering: 106 hospitals (> 60%) in Switzerland with STEMI/NSTEMI, voluntary participation Details: 33.040 patients (2010) Variables: 230 variables Funding: Private, sponsored by several industries
United States of America	NCDR®CathPCI (27, 28) National Cardiovascular Data Registry	1998	Covering: 1577 hospitals (90% of PCI-centres) in the United States Details: 12 million patients. Variables: 250 variables. Funding: reimbursement by insurance companies for participating hospitals

#### The relationship between registration of quality indicators and patient outcomes

Since registration is a time-consuming process, it is important to ascertain whether the used quality indicators actually provide the desired effect of improving quality of care. Chatterjee et al. have described three mechanisms by which registrations can help to improve patient outcomes [31].

First of all, *reporting about quality of care in cardiology itself* can lead to an incentive for hospital leaders and clinicians for improvement. In order to achieve this, it is important that results can be shared safely. Studies show that by paying attention internally to quality of care, improvement in outcomes of healthcare can be observed, which is called the Hawthorne effect [32].

*Public reporting* can also be a powerful incentive for clinicians and hospital leaders to improve. Besides, transparency increases confidence of patients in the healthcare system. However, public reporting of quality indicators in the United States also demonstrated some disadvantages. First of all, some studies comparing reporting states and non-reporting states show no differences in outcome [33]. A further concern of public reporting is that it will lead to risk aversion among physicians, deferring patients with more complex pathology, as is demonstrated in the literature. For example, in the United States, the majority (89 %) of interventional cardiologists have reported that the decision to intervene in critically ill patients was influenced by participating in the reporting of quality measures [33]. A registry confirmed this trend in practice, showing that patients in reporting states (e.g. New York) were less likely to undergo a PCI procedure if they were in shock [34]. Public reporting of CABG mortality in New York led to an increase of sicker patients being referred to the adjacent state Ohio [35]. Although transparency in quality indicators is increasing in the Netherlands, currently results cannot be linked to individual caregivers. The Society for Cardiothoracic Surgery in Great Britain and Ireland, in collaboration with the National Health Service, provides open access to information on treatment results of all individual cardiothoracic surgeons [36]. To improve transparency and to help patients in making informed decisions in the Netherlands, the aim of the Dutch government is to publish results of quality of care measurements at a national website for patients; www.kiesbeter.nl [3].

*Pay-for-performance* is the newest quality improvement mechanism, which is gaining attention from healthcare leaders and healthcare insurance companies as a strategy for maximising quality while controlling costs. Pay-for-performance implies a shift in paying for quality healthcare instead of volume of care, which can be a strong stimulus to improve quality [31, 37].

#### Pitfalls in quality measurement

As addressed before, the results of any measurement must be relevant for the different stakeholders in healthcare. In the current use and development of quality indicators it is also important to take into account that indicators are scientifically acceptable. The indicators should therefore be reliable and valid [38].

*Reliable* means that the indicator provides the same result on repeated measures and that the dataset is as complete as possible with uniform datasets which are collected in a uniform way. Also the Dutch Federation of University Medical Centres (Nederlandse Federatie van Universitair Medische Centra, NFU) points out in their report on a central vision on registration of care the value of a uniform standardised dataset. They aim to develop a uniform structure of elementary data elements and the use of a unified medical language based on international standards [39]. The use of universal definitions is encouraged by the International Consortium for Health Outcomes Measurement (ICHOM), an international non-profit organisation with the aim of transforming healthcare systems by measuring and reporting patient outcome [40].

*Validity* means that the indicator measures what it is intended to measure. This requires a good methodological quality, taking into account potential differences in case-mix and random variation. A common remark heard by doctors is that they worry about case-mix correction and that a negative outcome compared with others can be explained by the more difficult patient population served. A good case-mix correction applied in crude data could change the compared clinical outcome and is important to avoid unintended consequences [35, 41, 42]. Furthermore, it is important to remember that quality indicators are just an indication of the real quality of care. Therefore, the indicators should give appropriate coverage of the quality of care of a department and be in line with the crucial aspects of current strategies to improve quality of care.

#### Future challenges

On a national level, the current registries have to establish a unified collaboration and define useful indicators. NCDR, Meetbaar Beter and BHN are currently formalising this. It is intended that as of January 2016 NCDR and Meetbaar Beter will collaborate on the PCI database. On a regional level, hospitals should work together to acquire and exchange follow-up data, which requires commitment by the hospital’s board of directors in measuring quality of care on a local level. Possibly this can be achieved by appointing a board member on quality of care, as proposed by the Dutch government. Secondly, feasibility is of major importance. Currently, hospitals and their departments deliver between 600 and 1000 quality indicators to external parties each year. Since possibilities for automatic computation are still limited in most hospitals, this task is extremely time consuming. This leaves little time to use these externally reported quality indicators for internal improvement programs. Indicators should be easy to register in daily practice and ideally automatically obtainable. For this, integration of hospital electronic patient file systems with the national registries is essential. To ensure a long-term and reliable registry, solid funding is crucial. However, next to this national funding, sufficient funding on a departmental level is vital, especially if implantation is expected to become mandatory. Finally, it has to be decided what level of transparency is useful to improve the quality of care.

## Conclusion

The Dutch Minister of Healthcare has declared the year 2015 to be the year of transparency with the aim of improving healthcare by transparent reporting of quality of care. Furthermore, with the introduction of the regulated healthcare market, the position and role of the healthcare insurance companies provide an increasing focus on measuring quality of care. Initial thoughts are that quality indicators are connected with a lot of work without appreciating the benefits. Knowledge on and active participation in improving quality of care and quality measurements will be essential for cardiologists in daily clinical care. Active participation also offers major possibilities to design the most optimal quality measurement system and to take the lead in improving quality of care.

### Funding

None.

### Disclosures

Martin J. Schalij is a member of the Board of the National Cardiovascular Data Registry.

Bas A.J.M. de Mol is a member of the Board of Directors of Meetbaar Beter.

Giovanni Amoroso is a member of the Advisory Board of Meetbaar Beter.

Victor A.W.M. Umans is a member of the Board of Governors of the NCDR and Meetbaar Beter.
